# Serum Bile Acid Profiling and Mixed Model Analysis Reveal Biomarkers Associated with Pruritus Reduction in Maralixibat-Treated Patients with BSEP Deficiency

**DOI:** 10.3390/metabo12100952

**Published:** 2022-10-06

**Authors:** Xueheng Zhao, Wujuan Zhang, Pamela Vig, Cory Kostrub, Kenneth D. R. Setchell

**Affiliations:** 1 Division of Pathology and Laboratory Medicine, Cincinnati Children’s Hospital Medical Center, Cincinnati, OH 45229, USA; 2 Mirum Pharmaceuticals, Foster City, CA 94404, USA; 3 College of Medicine, University of Cincinnati, Cincinnati, OH 45229, USA

**Keywords:** bile acid metabolism, pruritus, linear mixed model, biomarker, maralixibat

## Abstract

Progressive familial intrahepatic cholestasis (PFIC) is a debilitating disease manifest by severe cholestasis, intractable pruritus and growth delay that ultimately leads to liver failure or transplantation. Maralixibat (MRX) was recently approved for the treatment of cholestatic pruritus in patients with Alagille syndrome. The aim of this study was to determine whether specific changes in the composition of the serum bile acid metabolome could predict pruritus response to treatment. Serum BAs (sBA) and 7α-hydroxy-4-cholesten-3-one (7α-C4), a surrogate marker of BA synthesis, were monitored by ultrahigh-performance liquid chromatography coupled with tandem mass spectrometry over 72 weeks in PFIC patients with mild to moderate non-truncating bile salt export pump (BSEP) mutations (*n* = 19) treated with MRX. The weekly itch reported outcome observer (ItchRO[Obs]) score measured pruritus severity. Linear mixed models (LMM) were applied to explore the effects of individual sBA profiles and their relationship to pruritus response. Changes in the composition of sBA correlated with pruritus improvement. Notably, the trajectory of serum total and individual BA species and 7α-C4 were significantly associated with ItchRO[Obs] score (*p* < 0.05). These results reveal that beyond simple total sBA concentrations, specific changes to the BA metabolome are associated with pruritus reduction in patients with BSEP deficiency, thus providing further insight into causal relationship of bile acids and pruritus.

## 1. Introduction

Progressive familial intrahepatic cholestasis type 2 (PFIC2) is a genetic disease resulting from the absence of, or reduction in functional activity of the bile salt export pump (BSEP) [1,2]. PFIC2 is caused by loss-of-function mutations in the *ABCB11* gene encoding BSEP and is inherited in an autosomal recessive manner. It causes severe cholestasis, elevations in serum bile acids, with subsequent intractable pruritus, growth retardation, liver injury requiring liver transplantation, and shortened life expectancy [2,3,4]. Compared to other progressive liver diseases with impairment of bile flow, such as Alagille syndrome, PFIC2 patients do not have hepatobiliary structural abnormality. Patients report that pruritus is often severe and leads to dermal damage through intense scratching and reduced health-related quality of life. The molecular mechanisms leading to cholestasis-associated pruritus are still unclear, and the involved pruritogens remain unknown. Since 1947, it was recognized that surgical biliary diversion, including nasobiliary drainage that provides immediate relief, attenuated pruritus, and bile acids, were long implicated, although not proven to be the causative agents in cholestatic pruritus [5,6]. Partial relief of pruritus after treatments with plasmapheresis, albumin dialysis, or plasma separation/anion absorption, suggest that the origins of the potential pruritogens are likely located in the systemic circulation [5].

Maralixibat (MRX) is a minimally absorbed, selective inhibitor of the ileal bile acid transporter (IBAT), which interrupts the enterohepatic circulation of bile acids (BAs), thereby reducing the highly elevated levels of circulating serum bile acids (sBAs) in cholestasis, and is shown to be effective in cholestatic patients with Alagille syndrome [7]. Maralixibat is currently Food and Drug Administration-approved for the treatment of cholestatic pruritus in patients with Alagille syndrome aged 1 year and older [8]. Results of a phase 2, open-label efficacy study in children with PFIC (INDIGO; NCT02057718) demonstrated reductions in sBA and pruritus in children with BSEP deficiency receiving MRX [9]. However, questions remain regarding the exact pathophysiology of cholestatic itch and the specific pruritogen that is modulated through IBAT inhibition by MRX to reduce itch.

Bile acids are synthesized from cholesterol in the liver. Primary bile acids, i.e., cholic acid (CA) and chenodeoxycholic acid (CDCA), and the glycine and taurine conjugates, are formed via two pathways, the neutral pathway and the acidic pathway [10]. Primary BAs are transformed by microorganisms in the small intestine into the principal secondary BAs, deoxycholic (DCA) and lithocholic (LCA). Detailed analysis of the serum BA (sBA) metabolome is critical to exploring the role of BA metabolism in cholestasis and pruritus, given that early studies indicate a role for bile acids underlying cholestatic itch, but later studies do not [11,12]. The molecular mechanisms leading to cholestasis-associated pruritus are still unresolved, and the specific pruritogens are at best indecisively identified [5]. Profiling the BA metabolome by improved mass spectrometry approaches should afford a better understanding of the pathological basis of pruritus, and the relevance of specific bile acid species in pruritus reduction, achieved to a greater extent in some PFIC2 patients than others treated with MRX. Moreover, the extended knowledge may be used to propose testable hypotheses for the exact mechanism of action of MRX in attenuating itch as it relates to altering the bile acid pool from baseline (BL).

The linear mixed model (LMM) algorithm has been successfully applied in the identification of genes or metabolites of interest in clinical research [13]. LMM is a flexible approach to allow the longitudinal data structure to be explicitly modelled. It can also accommodate for unbalanced longitudinal biomarker data, i.e., with missing time points for some patients in the study. Therefore, it is a powerful approach to apply to study the effects of sBA biomarkers on pruritus response of MRX treatment over time. LMM estimates the effects of one or more explanatory variables on a response variable. In this work, we investigated the sBA profile over time on pruritus response of patients undergoing MRX treatment by ultrahigh-performance liquid chromatography coupled with tandem mass spectrometry (UHPLC-MS/MS) and fitted LMM to discover the sBA biomarkers significantly correlated with pruritus reduction. The primary objective of this study was to evaluate changes in sBA including individual constituents from BL over 72 weeks (pre-dose baseline, 4, 8, 13, 48, 60, and 72 weeks) using the itch reported outcome score and to discover sBA biomarkers among children with pruritus response to MRX compared with non-responders. The secondary objective was to explore the role of the BA metabolism and related signaling pathway in pruritus response in PFIC2 patients.

## 2. Materials and Methods

### 2.1. Study Population

Enrolled patients were children (1–18 years of age) with genetically confirmed BSEP deficiency (biallelic ABCB11 mutations) treated with MRX (266 μg/kg/day initially [equivalent to maralixibat chloride 280 μg/kg, and hereafter referred to as 266 μg/kg], increased to 266 μg/kg twice daily if predefined sBAs and pruritus benefits were not achieved by week 72). All of these patients were receiving concomitant UDCA therapy (10–15 mg/kg/bw), which is a recommended standard of care therapy for these patients. An itch reported outcome (ItchRO) score was recorded by caregivers at baseline and during MRX treatment, and was the basis for the key pruritus endpoint. This was based on a 5-point scale (where 0 is no pruritus, 1 is mild, 2 is moderate, 3 severe, and 4 is very severe pruritus). A reduction in an ItchRO score of 1.0 or more were shown to be clinically meaningful [6]. In this study, pruritus treatment response was therefore defined as ≥ 1.0 reduction in the ItchRO(Obs) score at ≥ 1 time point. In total, 19 patients with mild to moderate non-truncating BSEP mutations were included in this analysis, 11 were classified as responders and 8 non-responders based on this score. In this study, “non-truncating” refers to the specific mutations and their effect on the transporter protein; mutations that are predicted to truncate the protein typically have a more severe effect on the protein function [14], so patients with two truncating mutations (one per chromosome pair) have the most severe disease, no bile flow, and are less likely to be helped by an IBAT inhibitor. We therefore focused on non-truncating mutations that have less severe disease and more bile flow. Additionally, response to treatment was defined by a sBA reduction of 70% from BL. In establishing this as an optimum threshold, we considered natural history data available in PFIC for improvement in sBA and their relation to longer term disease outcomes. Following a publication reporting improved transplant-free survival in individuals who achieved a reduction in sBAs > 75% from baseline or concentrations of <102.0 μmol/L after surgical interruption of the enterohepatic circulation by Van Wessel et al. [14], thresholds were changed to use this new definition in the post hoc responder analyses for this study.

Serum samples were obtained from all the enrolled patients pre-treatment (baseline) and at 4, 8, 13, 24, 48, 60, and 72 weeks post-treatment and stored at −80 °C until analysis. This study was approved by local institutional review boards, complied with the Declaration of Helsinki and Good Clinical Practice Guidelines, and was registered at Clinical Trials.gov, (accessed on 13 September 2022) (NCT02057718).

### 2.2. Analysis of Serum Bile Acids by Stable Isotope Diluted UHPLC-MS/MS

Quantitative analysis of individual and total serum bile acids was carried by stable-isotope dilution tandem mass spectrometry using a fully validated assay tandem mass spectrometry in a CAP/CLIA accredited laboratory (CCHMC). The major primary and secondary bile acids and their conjugates were quantified and these included, glycocholic acid (GCA), taurocholic acid (TCA), glycodeoxycholic acid (GDCA), taurodeoxycholic acid (TDCA), glycochenodeoxycholic acid (GCDCA), taurochenodeoxycholic acid (TCDCA), glycolithocholic acid (GLCA), taurolithocholic acid (TLCA), glycoursodeoxycholic acid (GUDCA), and tauroursodeoxycholic acid (TUDCA), and the unconjugated species CA, UDCA, CDCA, DCA, and LCA. The internal standards, a cocktail of 15 deuterium labelled standards (50 µL of a 1 ng/µL methanol solution) were added to serum (20 µL) and to the calibrators and QC serum samples. The samples were vortexed for 10 sec before addition of acetonitrile (200 µL). After vortex and centrifuge at 13,400× *g* at 4 °C for 10 min. The supernatant was removed and dried under nitrogen gas at 65 °C, and then reconstituted in 250 µL of 50% methanol/H_2_O and a 10 µL volume of this sample extract was injected on column for analysis by electrospray ionization LC-MS/MS using a Waters TQ-XS triple quadruple mass spectrometer interfaced with an Aquity UPLC system (Milford, MA, USA).

Individual bile acid species were separated by HPLC on a Kinetex C18 (2.6 µm, 100 × 3.0 mm) column (Phenomenex, Torrance, CA, USA) with gradient elution consisting of mobile phase A (20% acetonitrile/water with 10 mM ammonium acetate) and mobile phase B (80% acetonitrile/Water with 10 mM ammonium acetate). The total run time was 20 min. Serum bile acid concentrations are expressed as μmol/L and the total bile acid concentration is represented by the sum of the individual bile acid species measured. The bile acid composition is further expressed as a percent composition of the individual bile acid species. Total primary bile acids were calculated from the sum of GCA, TCA, CA, GCDCA, TCDCA, and CDCA; total secondary bile acids from the sum of GLCA, TLCA, LCA, GDCA, TDCA, DCA, GUDCA, TUDCA, and UDCA; total free bile acids from the sum of CA, CDCA, LCA, DCA, and UDCA; and total conjugated bile acids from the sum of glycine-conjugated and taurine-conjugated forms. The lower limit of quantification (LLOQ) for each BA species is the lowest standard that was selected with a response at least five times the blank response and a precision of 20% or less. Values less than LLOQ were replaced by half of the LLOQ value and used in the following statistical analysis.

### 2.3. Quantification of Serum 7α-Hydroxy-4-cholesten-3-one

7α-hydroxy-4-cholesten-3-one (7α-C4) was measured in plasma using a stable-isotope dilution LC-MS/MS assay as previously reported [15].

### 2.4. Statistical Analysis

Longitudinal profiling of total sBA and the trajectory of individual BA subspecies during MRX treatment was modelled with linear mixed effects models (LMM) to explore their association with pruritus response. Changes in total sBA and subspecies over 72 weeks of treatment with MRX (4, 8, 13, 48, 60, and 72 weeks) were compared with baseline and differences between pruritus responders and non-responders were examined. In the analysis, first we compared the sBA concentrations/pool at distinct time points during the treatment period. A two-tailed Student t-test was used and *p*-value < 0.05 indicates statistically significant differences. Pearson’s correlation was used to preliminarily explore the correlation levels between ItchRO score reduction and sBA species changes at each time point. All data are reported as mean ± standard error unless otherwise indicated. Since we were interested in the longitudinal change in sBA and individual subspecies implicated in the pruritus response, we used LMM to study the relationship among the sBA metabolic profile with cholestatic pruritus, as described below. All statistical analyses were conducted with the R language and environment for statistical computing (Version 4.1).

In building the LMM model, we first chose pruritus response (*ItchRO_score*) as treatment outcome. This is an intuitive choice, since it is a continuous variable and quantitatively reflects the severity of the pruritus. Longitudinal values of total serum bile acids concentration (µmol/L) at different time points were modelled for final parameter optimization. The composition of sBA subspecies in association with pruritus response were investigated. Data from all participants with samples from the available time points where all biomarker tests were measured were included in the models. As a first step in model fitting, initial age, gender, and baseline sBA concentration were fit as linear terms. A random intercept and slope was included for each patient and the default unstructured covariance structure for the residuals were chosen. We next fit this full model and tested the significance of covariates. Only covariates with *p* < 0.10 were retained in the final model. For the study of longitudinal sBA biomarkers, the following optimized model was applied to the final analysis:

(1) $$ItchRO\_score_{ij}=\left(\alpha_{1}+\beta_{i1}\right)+\left(\alpha_{2}+\beta_{i2}\right)t_{ij}+\beta_{1}BA_{i}+\delta_{ij}$$

With

(2) $$\beta_{i\;}=\left(\begin{array}{c} \beta_{i1}\\ \beta_{i2} \end{array}\right)\;∼\;N_{2}(\left(\begin{array}{c} 0\\ 0 \end{array}\right),\left(\begin{array}{c} \begin{array}{cc} D_{11} & D_{12} \end{array}\\ \begin{array}{cc} D_{21} & D_{22} \end{array} \end{array}\right))$$

where $\alpha_{1}$ represents the average intercept for the cohort, $\beta_{i1}$ represents the deviation of patient i from population average intercept, $\alpha_{2}$ represents the average slope/rate of change for the cohort, $\beta_{i2}$ represents the deviation of patient i from the population average slope. Covariance matrix **D** represents the variance of random effects $\beta_{i1}$ and $\beta_{i2}$, *D*_11_ is the variance of the individual intercepts $\beta_{i1}$, *D*_22_ is the variance of the individual intercepts $\beta_{i2}$, *D*_12_ and *D*_21_ equal to each other and are the covariance of the intercepts and slopes, $t_{ij}$ controls for time points (weeks), and $\delta_{ij}$ represents the standard deviation that is independent within patients and independent across patients for all individual patient and time points, i.e., $\delta_{ij}\;\sim \;N(0,\sigma^{2}$). Using this model, we can assess the effect of sBA biomarkers on the ItchRO(Obs) score longitudinally.

Because the pruritus response was defined by a reduction in ItchRO score ≥ 1.0, a separate general linear mixed effect model (GLMM) was also fit to explore each sBA biomarker identified by the previous LMM model. The general form of the GLMM model in matrix notation is:

(3) $$g\left(E\left(Y_{i}\right)\right)=X_{i}\beta +Z_{i}b_{i}$$

where ***Y***_*i*_ = (*Y*_*i*1_, *Y*_*i*2,_ … *Y_ini_*)^T^ is a vector of *n_i_* pruritus responses for patient *i*, i.e., pruritus responder if ≥1 reduction in the ItchRO score from baseline; ***X*** is the design matrix containing variables controlling for time and biomarkers; ***β*** is a column vector of the fixed-effects regression coefficients; ***Z***_*i*_ is the corresponding design matrix for the random effects (includes both intercept and time component); and ***b****_i_* is the random effects vector. In the model building, the link function, i.e., logistic regression was used to model the binary pruritus response variable. A general linear mixed model is a suitable method of choice for this setting, since measurement was taken repeatedly on the same participant over time during MRX treatment.

## 3. Results

### 3.1. Longitudinal Profiling of Serum BA and 7α-C4 by UHPLC-MS/MS

The serum BA pool including a concentration of individual BA species and the sterol intermediate 7α-C4, in PFIC patients, was followed longitudinally and measured by UHPLC-MS/MS. In total, the 15 major serum bile acids with a wide linear range for individual BA subspecies were quantified. The within-run and between-run reproducibilities, expressed as CV, of all measured BA subspecies at four different concentrations (LLOQ, low, medium, and high) were within 10%, respectively. Representative chromatograms of the sBA subspecies and 7α-C4 are illustrated in Supplemental Figures S1 and S2 (to add C4 chromatogram). Baseline characteristics and demographics of patients enrolled in this study are listed in Table 1.

**Figure 1:**
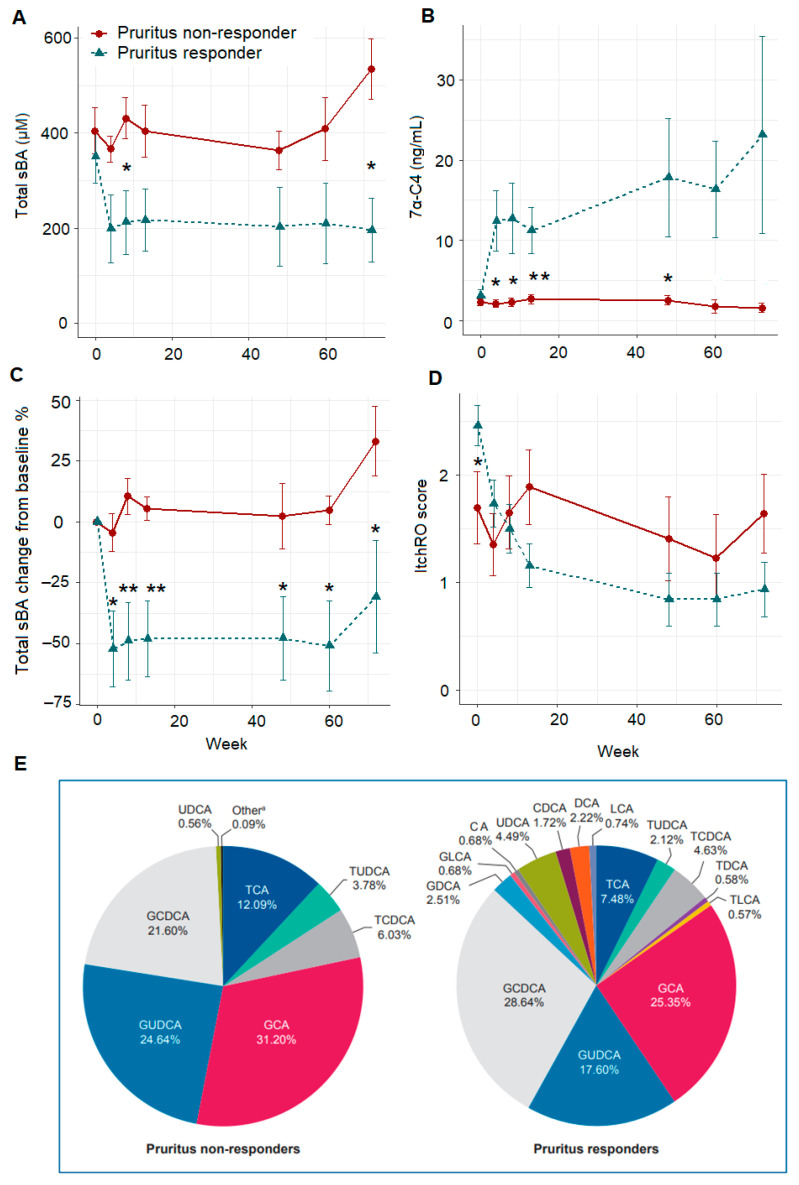
Longitudinal profile of total serum bile acid (**A**), 7α-C4 (**B**), total serum bile acid change from baseline (%) (**C**), and ItchRO score levels (**D**) over 72 weeks, i.e., baseline, 4, 8, 13, 48, 60, and 72 weeks of maralixibat treatment in pruritus responders vs. non-responders, data were represented as mean ± SE; composition of serum bile acid following 72 weeks of maralixibat treatment in pruritus responders vs. non-responders (**E**). * *p* ≤ 0.05, ** *p* ≤ 0.01. 7α -C4, 7 alpha -hydroxy-4-cholesten-3-one.

The trajectory shape of the total sBA, 7α-C4, change from baseline (%) in total sBA, and ItchRO score were investigated by a longitudinal plot where the vertical axis represents the value of each variable and the horizontal axis represents weeks of MRX treatment (Figure 1). After 72 weeks of treatment, PFIC2 patients with an improved ItchRO(Obs) score (pruritus responders) showed decreased total sBA concentrations compared with pruritus non-responders (mean, 195.97 ± 202.05 vs. 534.73 ± 155.40 μM, *p* = 0.004).

Serum 7α-C4 levels in pruritus responders increased during treatment, consistent with the biological action of MRX in inhibiting bile acid reuptake by the IBAT, increasing fecal BA excretion, and reducing the bile acid pool. In non-responders, 7α -C4 levels remained extremely low and relatively unchanged; concentrations were significantly lower when compared with pruritus responders throughout the treatment times. The percentage reduction in total sBA levels over 72 weeks revealed significantly greater changes in responders vs. non-responders for all time points (*p* ≤ 0.05). Furthermore, the greatest change in sBA composition (the average proportion of the total BA pool for each BA subspecies) after 72 weeks of MRX treatment was observed in the pruritus responder group. By contrast, changes in the non-responder group were minimum (Figure 1E). Longitudinal changes in the proportion of the sBA subspecies was thus explored over the study period by a similar longitudinal plot, where the vertical axis represents the composition (%) of each BA species and the horizontal axis represents weeks of MRX treatment (Supplemental Figures S3 and S4). The proportional relationships among sBA subspecies, including unconjugated and conjugated BAs, were also plotted to detect any differences between responder groups (Supplemental Figure S5).

A trend toward increased proportions of unconjugated sBAs was observed in the pruritus responders (9.84 ± 4.87%) vs. non-responders (0.61 ± 0.24%, *p* = 0.09). These results suggest there is either some degree of restoration of bile flow after MRX treatment, because unconjugated serum bile acids are derived from the intestinal bacterial hydrolysis of primary bile acids secreted into bile, or alternatively, the increased loss of primary bile acids into the colon results in deconjugation and 7α-dehydroxylation to secondary BA by colonic microbiota. Trajectory of sBA subspecies composition strengthens the overall trend at 72 weeks, i.e., GCA (%) decreased and GCDCA (%) increased over time, and in general, unconjugated sBA and secondary BA increased with MRX treatment. TCA and GCA demonstrated the greatest reductions in sBA pool (43.3 ± 21.2% and 32.1 ± 26.2% reductions, respectively, baseline vs. week 72) in pruritus responders (Table 2).

**Figure 2:**
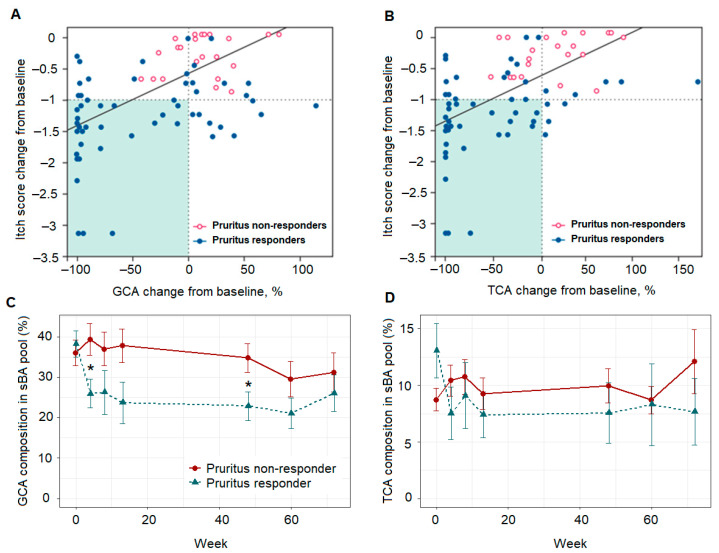
Correlation between change from baseline of conjugated serum bile acid species GCA, Pearson’s r 0.549 (*p*-value < 0.001) (**A**) and TCA, Pearson’s r 0.605 (*p*-value < 0.001) (**B**), and reduction in the ItchRO(Obs) score following 72 weeks of maralixibat treatment, correlation coefficients were calculated using full patient cohort. Background highlighted in green indicates reduction in both ItchRO(Obs) score and measured sBA % change from baseline. GCA, glycine-conjugated cholic acid; ItchRO(Obs), itch reported outcome score from observer; sBA, serum bile acid; and TCA, taurine-conjugated cholic acid. Two subjects are excluded due to baseline ItchRO(OBs) scores < 1.0. Profile of GCA (**C**) and TCA (**D**) following 72 weeks of maralixibat treatment. * indicates significant *p*-value, i.e., at 0.05 level, between pruritus responders vs non-responders at a given time point.

Reductions in GCA and TCA due to MRX treatment correlated with pruritus reduction in responders (Pearson: 0.55 and 0.61 for GCA and TCA, respectively; both *p* < 0.001 vs. non-responders; Figure 2). A general decreased trajectory of both GCA and TCA was observed over 72 weeks of treatment (Figure 2, Supplemental Table S1). However, there was limited power to detect the association of longitudinal sBA profiles and pruritus reduction at the patient level. To overcome this issue, we applied the linear mixed effect model (LMM) to explore longitudinal changes in BA species in cholestatic pruritus.

### 3.2. Pruritus ItchRO Score as Continuous Variable in Building LMM Model

To model the relationship between BA metabolic biomarkers in serum to pruritus change, we chose pruritus reduction over the study period, i.e., 72 weeks, as the response variable. First ItchRO(Obs) score was evaluated, which is a continuous outcome variable, and recorded at seven time points, i.e., pre-dose baseline, 4, 8, 13, 48, 60, and 72 weeks during MRX treatment. The effects of the total sBA (i.e., reduction in the total pool via MRX treatment) and sBA metabolome (composition of sBA subspecies) on the ItchRO(Obs) score was examined by longitudinal data analysis. In initial model fitting, age of the patient at the treatment start, gender, and baseline sBA concentration of participants were fit as linear terms. Random effect was determined based on the experimental design, i.e., the individual patient under MRX treatment.

**Figure 3:**
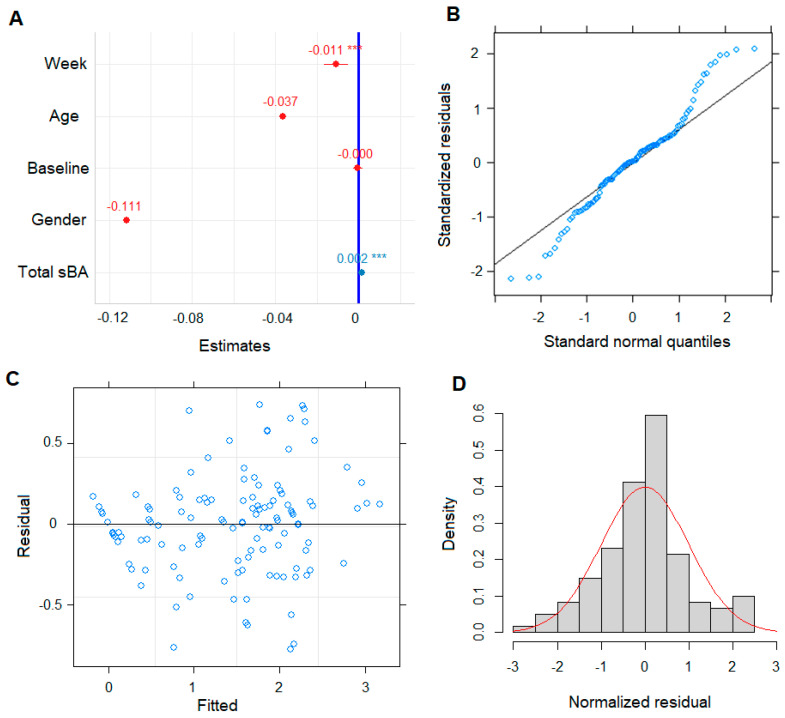
Evaluation of total sBA as a biomarker (predictor) in building the LMM model. Coefficients of fixed factors/effects, i.e., total sBA concentration during the study period, weeks of patients on MRX in study, initial age and gender of patients, and sBA pre-dose baseline from LMM model, whiskers span the 95% CI, *** *p*-value < 0.001, color of a dot indicate the factor/covariate has negative (red) or positive (blue) effects on ItchRO score (**A**); QQ-plot of residuals for normality check (**B**); residual plot against the fitted values for homoscedasticity (**C**); and histogram of normalized residuals (**D**).

To maximize random effect structure, both random intercept and slope of within-subject effects were chosen. This comprehensively built model converged and the residual plot did not indicate any deviations from a linear form (Figure 3). Evidence of data compliance was assessed on assumption of the linear mixed model, i.e., homoscedasticity and normality of the residuals. The QQ-plot and histogram of residuals showed minor departure from normality, but there was no major deviation observed (Figure 3). Residuals were also plotted against the fitted values from the model and there is no obvious heteroscedasticity in the data detected (Figure 3). We plotted the residual based on the gender of the patients, pruritus responder status, age, and individual patient model fitted ItchRO score (Supplemental Figure S6). In fitting the full model to test for significance, neither of the covariates, i.e., age, gender, and pre-dose baseline, were significant with *p* < 0.10 (Table 2). A further likelihood ratio test compared the reduced model, i.e., excluding these non-significant variables from the full model, indicating the reduced model better fitted the data with lower Akaike information criterion (AIC) and Bayesian information criterion (BIC) (Supplemental Table S2). An insignificant *p*-value (i.e., 0.63) indicated these three covariates did not have significant effects on ItchRO(Obs) score (Supplemental Table S3). Therefore, a simpler model without these fixed effects was chosen throughout the rest of the analysis on studying effects of BA subspecies composition and metabolism.

### 3.3. LMM Revealed sBA Pool Associates with Patient Pruritus Response

Given that individual BA species showed different trajectories in pruritus phenotype manifestation over time, the LMM model was used to explore the relationship between longitudinal sBA species profile and pruritus reduction. In the model fitting, ItchRO(Obs) score was modelled as an outcome variable. Each BA species proportion of the total BA pool was modelled as fixed effect in the LMM. Patients were again modelled as random effect variables with a different intercept and slope. Then the relationship between the sBA subspecies and the response variable, i.e., ItchRO(Obs) score using the LMM model, could be estimated.

**Figure 4:**
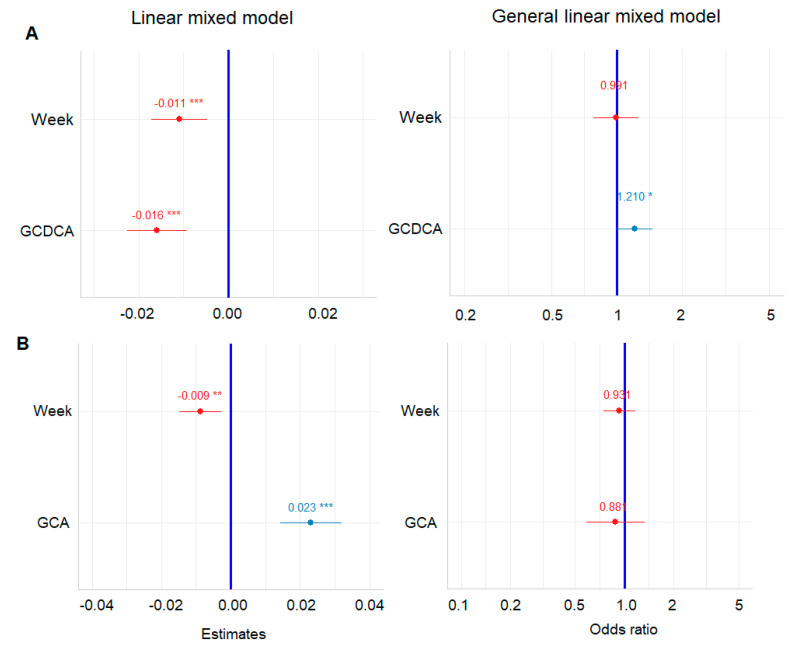
Estimates of coefficient and odds ratio of GCDCA composition (%) (**A**) and GCA composition (%) (**B**) in the sBA pool as biomarker (predictor) in LMM and GLMM models, respectively. * *p*-value ≤ 0.05, ** *p*-value ≤ 0.01, *** *p*-value < 0.001. Color of dot indicate the factor/covariate has negative (red) or positive (blue) effects on ItchRO score in LMM model, and responder status, i.e., non-reponder 0 and responder 1, in GLM model; Whiskers span the 95% confidence interval, GCDCA, glycochenodeoxycholic acid; and GCA, glycocholic acid.

Out of the 15 BA species, the GCDCA coefficient estimate showed a negative value of −0.02 (−0.02, −0.01; 95% CI) with a *p*-value <0.001 (Figure 4). This indicates a significant negative relationship for the GCDCA composition and the ItchRO(Obs) score as shown in Table 3. Longitudinal data plot based on pruritus response also showed the opposite trend between the two groups, i.e., pruritus responder vs. non-responder (Supplemental Figure S7). The other BA species that showed negative relation to ItchRO(Obs) score with a significant *p*-value included UDCA, with an estimate of −0.03, *p*-value 0.002 (Supplemental Table S4). By contrast, several BA species showed significant positive relation to ItchRO(Obs) score in the longitudinal manner; GCA, TCA, with an estimate of 0.02, 0.04, and *p*-value <0.001, <0.001, respectively (Supplemental Tables S5 and S6). Furthermore, the ratios of GCA/GCDCA, TCA/TCDCA, total cholic acids/total chenodeoxycholic acids, and unconjugated BA/conjugated BA were also examined in the LMM model. The estimate of the fix effect was 0.21, 0.25, 0.23, and −1.62 with *p*-value < 0.001, < 0.001, < 0.001, and < 0.001, respectively. On average, patients have a 0.23-units-higher ItchRO(Obs) score when total CA/total CDCA changes one unit. Unconjugated BA/conjugated BA ratio showed a clear negative relationship to ItchRO(Obs) score. These results imply a strong relationship between the BA subspecies/metabolism and the pruritus reduction in PFIC2 patients under MRX treatment instead of just the total sBA levels.

### 3.4. Pruritus Response Class Variable as Binary Outcome

To confirm the robustness and accuracy of effect estimates in model building and data preparation, a generalized linear mixed model (GLMM) was used as an alternative way to validate and model this dataset. In the GLMM, patients were dichotomized by the pruritus responders (*n* = 11), those with ≥1 reduction at one or more than one time points, vs. non-responders (*n* = 8), who were those with ItchRO score changes of less than 1 during the 72-week period. In the model, pruritus non-responders and responders were dummy coded as 0 and 1. The GLMM model showed that the GCDCA estimate (odds ratio) was a positive value of 1.21 (1.00, 1.46; 95% CI) with a *p*-value 0.046 (Table 3). This implicates that, on average, the patient has a 21% increase in the chance of being a pruritus responder when GCDCA (%) has one unit increase. This confirmed that GCDCA composition relates to pruritus reduction in patients under MRX treatment. As expected, GCA (%) and the ratio of the total cholic/chenodeoxycholic acids showed a negative relationship to pruritus responders with an estimate odds ratio of 0.88 and 0.22, respectively. However, the *p*-value did not reach significance (Supplemental Tables S7 and S8). The ratio of unconjugated BA/conjugated BA failed to converge in the GLMM model, therefore, the result was not included herein. Based on the results from both LMM and GLMM models, it was unambiguously demonstrated that the BA pool, i.e., BA metabolism change, associates with pruritus response in PFIC patients treated with MRX.

**Figure 5:**
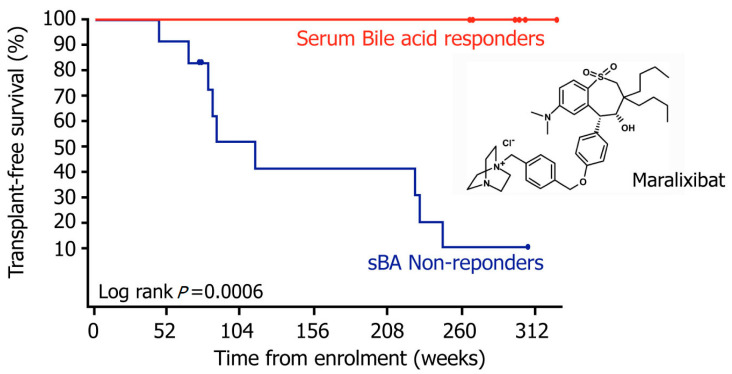
Median overall survival rate among the PFIC2 patients. This graph shows the median overall transplant-free survival rate among the PFIC2 patients, using Kaplan–Meier curve analysis, for those patients that had a significant sBA decrease, designated as sBA responders (red), compared with those patients that showed an insignificant sBA change, designated as sBA non-responders (blue). The *X*-axis represents weeks and the *Y*-axis represents the percentage of patients that were transplant-free.

Further evidence of long-term treatment benefit was evident from the transplant-free survival measured up to 312 weeks. Those patients that exhibited a significant reduction in sBA in response to maralixibat treatment, were transplant-free up to 312 weeks. These sBA responders also experienced a significant improvement in pruritus, serum transaminases, bilirubin, quality of life as well as in growth parameters [9] (Figure 5).

## 4. Discussion

Although the pathogenesis of cholestasis-associated pruritus is still poorly understood, we hypothesized that changes in BA biosynthesis/metabolism, other than just total sBA changes, associates with, and is predictive of, pruritus reduction. Therefore, based on response to MRX therapy, we explored longitudinal serum BA profiles, i.e., the composition of sBA trajectory, in response to the pruritus reduction in PFIC2 patients treated with MRX.

**Figure 6:**
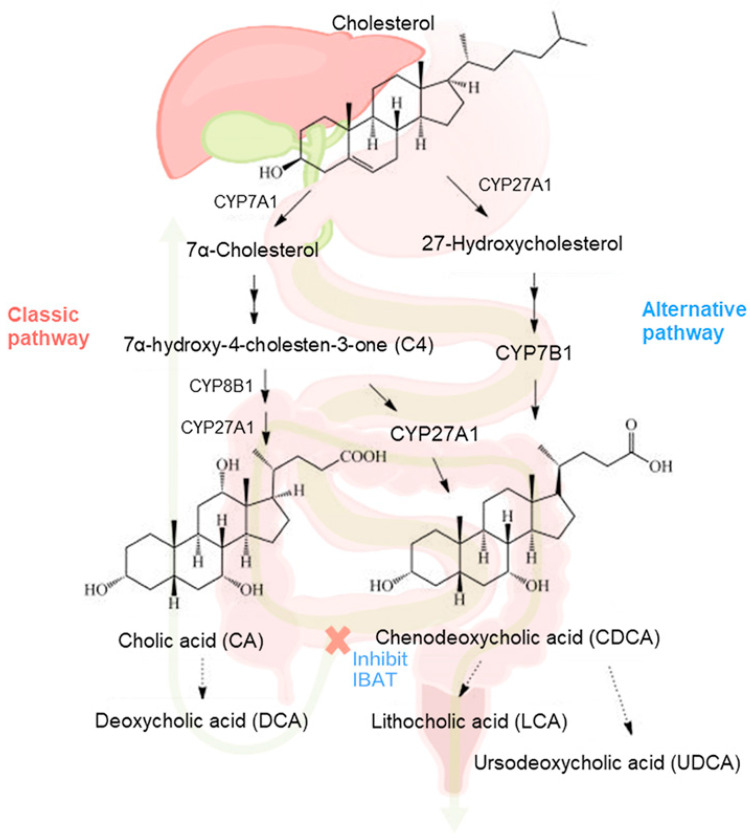
Illustration of BA metabolism in liver and intestine under MRX treatment.

Bile acids are synthesized from cholesterol in the liver by a complex pathway that is regulated by the rate-limiting enzyme cholesterol 7α-hydroxylase (CYP7A1) (Figure 6). The sterol intermediate 7α -C4 is the key precursor in the neutral or classical pathway for the synthesis of the primary bile acids CDCA and CA. Sterol 12α-hydroxylase (CYP8B1) directs synthesis toward cholic acid by 12α-hydroxylation of 7α -C4. The final step in the pathway is efficient conjugation to the amino acids glycine and taurine, so that unconjugated bile acids account for <1% of the total bile acids in bile. Primary bile acids are transported into bile via the bile salt export pump (BSEP) transporter protein localized on the canalicular membrane. In the intestine, primary bile acids are first deconjugated by bile acid hydrolases and then 7α-dehydroxylated by gut bacteria to form the secondary bile acids deoxycholic and lithocholic acids (Figure 6). Changes in the bile acid metabolome may therefore have differing effects on cholestatic pruritus through pruritus-mediating receptors, including the G-protein-coupled bile acid receptor, Gpbar1 (TGR5) and farnesoid X receptor (FXR) [16]. Bile salts are proposed to mediate their pruritic effects through TGR5, expressed on sensory neurons, and the nuclear transcription factor FXR, present in extraohepatic tissues, skin, and brain neurons. Bile salts bind to these receptors, activating signaling cascades and transcriptional networks intracellularly, which is thought to contribute to pruritus [17]. Since patients who increased GCDCA tend to respond to MRX and have pruritus relief, it is possible that antagonizing FXR potentially accounts for the cholestatic pruritus relief. Furthermore, in this study, changes in serum GCA and TCA were associated with the improved pruritus in PFIC2 patients.

Over the past 60+ years, the role bile acids play as causative agents for the pruritus associated with liver disease was debated. The observation that biliary drainage relieved pruritus pointed to a probable pruritogen in bile, and bile acids were considered candidates because they were elevated in patients with pruritus. Topical administration of bile acids, prick, and patch tests [18] were largely unconvincing, but unconjugated bile acids were claimed to cause itching when applied to skin blisters, and analysis of swabbed skin surfaces suggested dihydroxy, in particular deoxycholic acid was present in higher levels in pruritic patients than non-pruritic patients. Thus detailed analysis of the bile acid metabolome is important to better understand the etiology of itch. Later studies of serum and skin tissue and interstitial fluid did not provide convincing evidence that bile acids were causative, and the search for other agents continued, with opioids, progesterone sulfates, and autotoxin and lysophosphatidic acid all proposed to play a role.

Monitoring the effects of treatment using updated biomarkers that are measured routinely over time can be an important part of patient care. The change in biomarkers over time is considered as an indicator of disease state/response status and is helpful for dose optimization. In this work, we studied the profile of serum bile acids at various time points, including pre-MRX and during MRX treatment, to explore BA metabolic changes in pruritus response and to try to explain why some patients responded to treatment while others did not. From the modeling, we identified sBA biomarkers related to pruritus response. The composition of serum bile salts varied differently longitudinally among patients who responded to treatment with pruritus reduction and those who did not. The updated total CA/CDCA ratio had the highest fixed effect on ItchRO(obs) score, indicating that it is a potential biomarker for practical use. More importantly, it implied that BA metabolism improvement is associated with the pruritus response in these PFIC patients.

One limitation of this study is that all patients were prescribed ursodeoxycholic acid (UDCA) in the course of their typical clinical care. UDCA could not be justifiably stopped and consequently UDCA therapy continued after initiating and during MRX treatment. This requires consideration when interpreting the bile acid composition, because UDCA is partially metabolized to CDCA and this will be reflected in the sBA profile (i.e., the relative concentration of each of the BA subspecies). It likely explains the increase in serum levels of GCDCA. Nevertheless, despite all patients being on UDCA therapy, significant differences were observed in the sBA composition between pruritus responders when compared with non-responders. The changes in sBA pool revealed specific BA as biomarkers associated with pruritus reduction in MRX treatment. To impute values below the limit of quantification (BLOQ), we used half of the LLOQ value. As a simple and pragmatic solution, we believe that it is more accurate than setting the BLOQ values to 0, since the level of endogenous BA species, even if unmeasurable, definitely exists in serum samples. The disadvantage of this approach is that it produced an arbitrary variance/standard deviation for some extremely low concentration BA species, including LCA, TLCA, and GLCA (Supplemental Table S1). We recognized this limitation in our data analysis and were conservative on data interpretation to avoid bias. Importantly, the effect of BLOQ value imputation on total BA and most individual BA species with moderate to high concentrations was minimal.

The mechanism by which MRX leads to a reduction in pruritus severity is likely due to the reductions in sBA due to the mechanism of action targeting bile acid accumulation, however, the specific compositional differences in bile acids that are seen between responders and non-responders to MRX therapy are complex. It is interesting that a drug so minimally absorbed, and one that blocks the reuptake of bile acids from the intestine, has such profound effects on hepatic bile acid synthesis and metabolism. Increases in serum 7α-C4, a surrogate marker for CYP7A1 activity, provide convincing evidence that hepatic bile acid synthesis is restored by MRX. Serum 7α-C4 concentrations, a surrogate marker for bile acid synthesis rate, returned to normal levels after treatment in responders, but remained at extremely low concentrations in those patients that had no pruritus relief.

The increase in the serum composition of unconjugated bile acids is also highly significant. Serum unconjugated bile acids reflect the balance between intestinal input, hepatic extraction, and hepatocyte efflux across the basolateral membrane, the latter being significant in cholestasis. High serum unconjugated and secondary bile acids are a sensitive marker for small bowel bacterial overgrowth [19], while negligible concentrations in serum are a feature of cholestasis. In the pruritus non-responders, unconjugated bile acids, and especially the secondary bile acids of DCA and LCA, were either absent or present in only traces, consistent with the lack of the secretion of bile into the intestine. By contrast, in the pruritus responders, there were significant and normal concentrations/proportions of unconjugated bile acids in serum after MRX treatment. This seemingly can only be explained by an improvement in bile flow with MRX treatment, and not by increased loss of primary bile acids into the colon due to IBAT inhibition, with subsequent bacterial metabolism. If the source of these unconjugated bile acids was colonic in origin, then there would be no differences in the proportions of unconjugated bile acids in both responders and non-responders, but this was not the case. A further possibility might be that MRX alters the microbiome, but that was not investigated in this study. Furthermore, it should be mentioned that unconjugated bile acids are absorbed from the small intestine and colon [20] by non-ionic passive diffusion and do not require active transport through IBAT. Thus MRX would not prevent their appearance in serum. It would also not interfere with UDCA therapy, which was consistent in all patients throughout the study.

## 5. Conclusions

Our findings reveal for the first time that the pruritus response in children with non-truncating BSEP deficiency, administered MRX was associated with significant compositional changes in sBA. Reductions in sBA, GCA, and TCA, along with concomitant increases in the sterol intermediate 7α-C4 and the proportion of GCDCA, were associated with pruritus relief. Our findings show that when MRX modifies the BA metabolome, it leads to significant reductions in the severity of pruritus in PFIC2 patients. This minimally absorbed drug that targets the key transporter for maintaining the enterohepatic circulation of bile acids also has profound effects on hepatic bile acid synthesis and metabolism. These findings offer new insights into BA subspecies changes associated with reductions in cholestatic pruritus, reinforcing data demonstrating that BA metabolism changes underlie the improvement of itch in PFIC2 patients treated with MRX, which ultimately was reflected in transplant-free survival.

## Figures and Tables

**Table 1:** Baseline characteristics of PFIC patients with prognostic groups in pruritus response under MRX treatment *.

Characteristics	All	Pruritus Responder	Pruritus Non-Responder
Number of participants	19	11	8
Age (at enrollment), y, median (IQR)	3 (1.5, 4.5)	3.0 (1, 4.5)	3.5 (2.8, 5.5)
Female No. (%)	68	73	63
Pruritus score, median (IQR)	1.9 (1.9, 2.1)	2.5 (1.9, 3.0)	1.9 (1.5,2.0)
Mutation	Mild/Moderate (7/12)	Mild/Moderate (4/7)	Mild/Moderate (3/5)
Total BA (µM), median (IQR)	423 (286, 478)	423 (238, 440)	437 (303, 501)
7α-C4 (ng/mL), median (IQR)	2.3 (1.1, 3.8)	1.9 (1.1, 4.8)	2.5 (2.0,3.0)
FGF 19 (pg/mL), median (IQR)	181 (107, 810)	116 (84,568)	687 (171,993)
Urso supplement (n)	19	11	8

* Difference in each characteristic was evaluated by Welch’s two-sample *t* test. *p* > 0.05 for all baseline characteristics between pruritus responders and pruritus non-responders.

**Table 2:** Summary of linear mixed model (LMM) of ItchRO score ~ total sBA (uM).

	ItchRO Weekly Average Score		
Predictors	Estimates	CI	*p*-Value
(Intercept)	1.49	0.47–2.51	0.005 *
Week	−0.01	−0.02–−0.01	**<0.001**
Age	−0.04	−0.14–0.07	0.479
Pre-dose baseline (sBA)	−0.0003	−0.00–0.00	0.779
Gender	−0.06	−0.42–0.31	0.761
Total sBA	0.002	0.00–0.00	**<0.001**
N _Subject_	19		
Observations	121		

* Significant *p*-values, i.e. at 0.05 level, are highlighted in bold.

**Table 3:** Summary of the linear and general linear mixed models (LMM and GLMM) of ItchRO score ~ GCDCA (%).

	ItchRO Weekly Average Score			Pruritus Responder		
Predictors	Estimates	CI	*p*-Value	Odds Ratios	CI	*p*-Value
(Intercept)	2.17	1.80–2.54	**<0.001**	0.00	0.00–0.00	**0.008**
Week	−0.01	−0.02–−0.00	**0.001**	0.99	0.78–1.26	0.940
GCDCA	−0.02	−0.02–−0.01	**<0.001**	1.21	1.00–1.46	**0.046**
N	19 _Subject_ID_			19 _Subject_ID_		
Observations	121			121		

Significant *p*-values, i.e. at 0.05 level, are highlighted in bold.

## Data Availability

All generated data are either summarized or represented to support the findings of this work and available within the article.

## Supplementary Materials

The following supporting information can be downloaded at: https://www.mdpi.com/article/10.3390/metabo12100952/s1, Figure S1. Serum bile acid profiling by UHPLC-MS/MS, total 15 BA subspecies quantified in the established assay; Figure S2. Representative chromatogram of serum C4 profiling by UHPLC-MS/MS. Figure S3. Change in conjugated serum bile acid composition (%) over 72 weeks of maralixibat treatment in pruritus responders vs. non-responders. Figure S4. Change in unconjugated serum bile acid composition (%) over 72 weeks of maralixibat treatment in pruritus responders vs. non-responders; Figure S5. Change in serum bile acid composition (%) relationship (ratio) over 72 weeks of maralixibat treatment in pruritus responders vs. non-responders. Figure S6. Residuals from LMM model; Figure S7. Profile of GCDCA composition (%) over 72 weeks in PFIC2 patients under MRX treatment; Table S1. Levels of serum bile acid in patients with non-truncating BSEP following 72 weeks of maralixbat treatment compared with baseline; Table S2. Total BA as biomarker—model optimization; Table S3. Likelihood-ratio test for reduced model excluding age, gender, and pre-dose baseline vs. full model; Table S4. Summary of linear mixed model (LMM) of pruritus ItchRO score ~ UDCA (%); Table S5. Summary of linear mixed model (LMM) of pruritus ItchRO score ~ GCA (%); Table S6. Summary of linear mixed model (LMM) of pruritus ItchRO score ~ TCA (%); Table S7. Summary of general linear mixed model (GLMM) of pruritus responder class ~ GCA (%); Table S8. Summary of general linear mixed model (GLMM) of pruritus responder class ~ ratio of total cholic acids/total chenodeoxycholic acids.

## References

[1] Malatack J.J., Doyle D. (2018). A Drug Regimen for Progressive Familial Cholestasis Type 2. Pediatrics.

[2] Baker A., Kerkar N., Todorova L., Kamath B.M., Houwen R.H.J. (2019). Systematic review of progressive familial intrahepatic cholestasis. Clin. Res. Hepatol. Gastroenterol..

[3] Jones-Hughes T., Campbell J., Crathorne L. (2021). Epidemiology and burden of progressive familial intrahepatic cholestasis: A systematic review. Orphanet J. Rare Dis..

[4] Srivastava A. (2014). Progressive familial intrahepatic cholestasis. J. Clin. Exp. Hepatol..

[5] Langedijk J., Beuers U.H., Oude Elferink R.P.J. (2021). Cholestasis-Associated Pruritus and Its Pruritogens. Front. Med..

[6] Hegade V.S., Krawczyk M., Kremer A.E., Kuczka J., Gaouar F., Kuiper E.M., van Buuren H.R., Lammert F., Corpechot C., Jones D.E. (2016). The safety and efficacy of nasobiliary drainage in the treatment of refractory cholestatic pruritus: A multicentre European study. Aliment. Pharmacol. Ther..

[7] Gonzales E., Hardikar W., Stormon M., Baker A., Hierro L., Gliwicz D., Lacaille F., Lachaux A., Sturm E., Setchell K.D.R. (2021). Efficacy and safety of maralixibat treatment in patients with Alagille syndrome and cholestatic pruritus (ICONIC): A randomised phase 2 study. Lancet.

[8] Mirum Pharmaceuticals (2021). Livmarli™ (Maralixibat) Oral Solution.

[9] Loomes K.M., Squires R.H., Kelly D., Rajwal S., Soufi N., Lachaux A., Jankowska I., Mack C., Setchell K.D.R., Karthikeyan P. (2022). Maralixibat for the treatment of PFIC: Long-term, IBAT inhibition in an open-label, Phase 2 study. Hepatol. Commun..

[10] Javitt N.B. (1994). Bile acid synthesis from cholesterol: Regulatory and auxiliary pathways. FASEB J..

[11] Kuiper E.M., van Erpecum K.J., Beuers U., Hansen B.E., Thio H.B., de Man R.A., Janssen H.L., van Buuren H.R. (2010). The potent bile acid sequestrant colesevelam is not effective in cholestatic pruritus: Results of a double-blind, randomized, placebo-controlled trial. Hepatology.

[12] Ghent C.N., Bloomer J.R., Klatskin G. (1977). Elevations in skin tissue levels of bile acids in human cholestasis: Relation to serum levels and topruritus. Gastroenterology.

[13] Wanichthanarak K., Jeamsripong S., Pornputtapong N., Khoomrung S. (2019). Accounting for biological variation with linear mixed-effects modelling improves the quality of clinical metabolomics data. Comput. Struct. Biotechnol. J..

[14] van Wessel D.B.E., Thompson R.J., Gonzales E., Jankowska I., Sokal E., Grammatikopoulos T., Kadaristiana A., Jacquemin E., Spraul A., Lipinski P. (2020). Genotype correlates with the natural history of severe bile salt export pump deficiency. J. Hepatol..

[15] Zhao X., Setchell K.D.R., Huang R., Mallawaarachchi I., Ehsan L., Dobrzykowski Iii E., Zhao J., Syed S., Ma J.Z., Iqbal N.T. (2021). Bile Acid Profiling Reveals Distinct Signatures in Undernourished Children with Environmental Enteric Dysfunction. J. Nutr..

[16] Chiang J.Y. (2017). Recent advances in understanding bile acid homeostasis. F1000Research.

[17] Hussain A.B., Samuel R., Hegade V.S., Jones D.E., Reynolds N.J. (2019). Pruritus secondary to primary biliary cholangitis: A review of the pathophysiology and management with phototherapy. Br. J. Dermatol..

[18] Kirby J., Heaton K.W., Burton J.L. (1974). Pruritic effect of bile salts. Br. Med. J..

[19] Setchell K.D., Harrison D.L., Gilbert J.M., Mupthy G.M. (1985). Serum unconjugated bile acids: Qualitative and quantitative profiles in ileal resection and bacterial overgrowth. Clin. Chim. Acta.

[20] Dawson P.A., Karpen S.J. (2015). Intestinal transport and metabolism of bile acids. J. Lipid Res..

