# Supplementary material for: Serum Bile Acid Profiling and Mixed Model Analysis Reveal Biomarkers Associated with Pruritus Reduction in Maralixibat-Treated Patients with BSEP Deficiency
Source: Metabolites. 2022 Oct 6;12(10):952. doi: 10.3390/metabo12100952 (PMC9609791; doi:10.3390/metabo12100952)
Supplement: Supplementary file 1 [file metabolites-12-00952-s001.zip › Supplemental_figures.pdf]

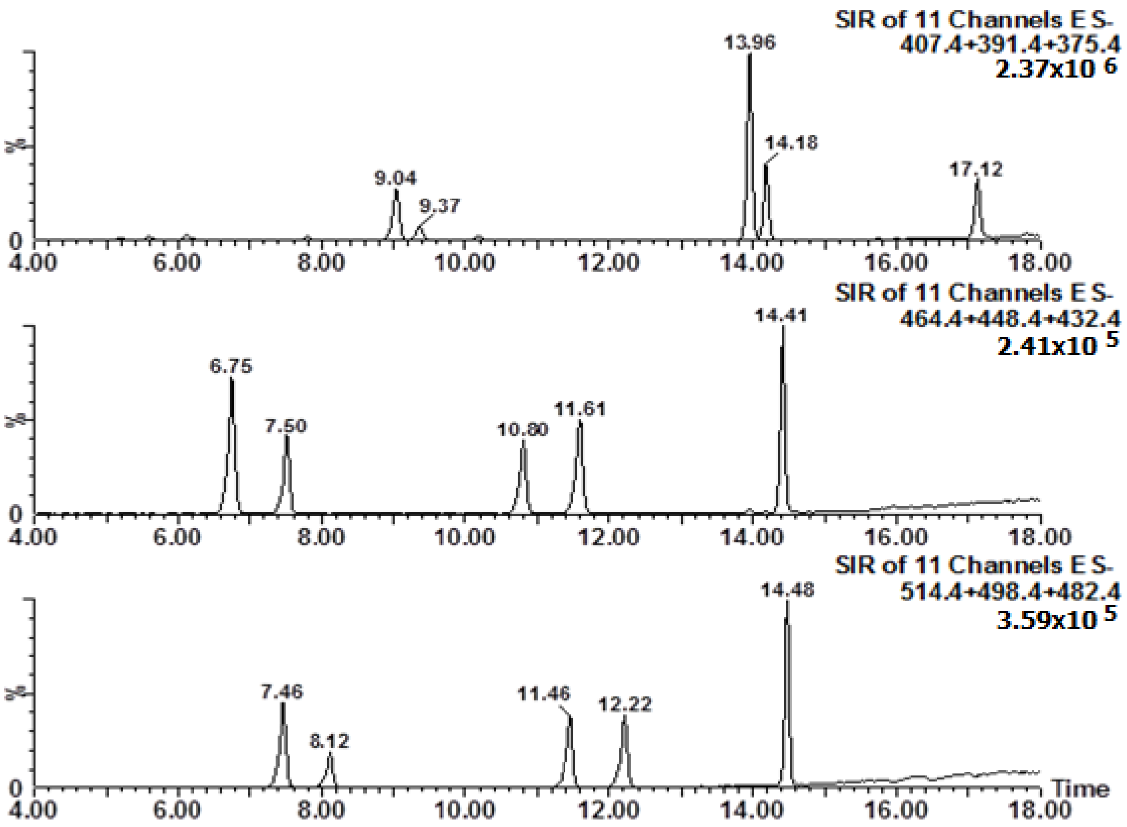
SIR of 11 Channels E S-
407.4+391.4+375.4
2.37x10 6
13.96
14.18
17.12
9.04
9.37
%
0
4.00
6.00
8.00
10.00
12.00
14.00
16.00
18.00
SIR of 11 Channels E S-
464.4+448.4+432.4
2.41x10 5
14.41
6.75
7.50
10.80
11.61
%
0
4.00
6.00
8.00
10.00
12.00
14.00
16.00
18.00
SIR of 11 Channels E S-
514.4+498.4+482.4
3.59x10 5
14.48
7.46
8.12
11.46
12.22
%
0
Time
4.00
6.00
8.00
10.00
12.00
14.00
16.00
18.00

(**a**)

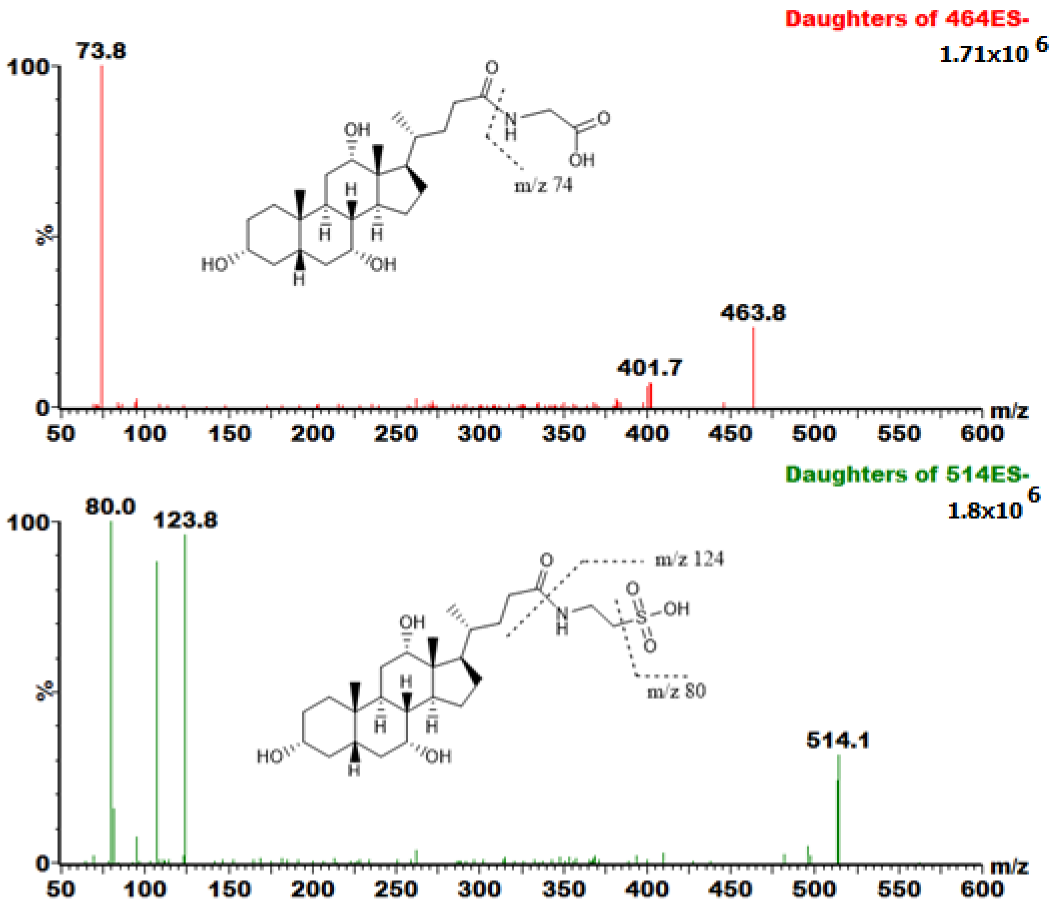

(**b**)

Supplemental Figure S1. (**a**) Serum bile acid profiling by UHPLC-MS/MS, total 15 BA subspecies quantified in the established assay. CHA, cholic acid; CDCA, chenodeoxycholic acid; DCA, deoxycholic acid; LCA, lithocholic acid; GCA, glycine-conjugated cholic acid; GCDCA, glycochenodeoxycholic acid; GDCA, glycodeoxycholic acid; GLCA, glycolithocholic acid; GUDCA, glycoursodeoxycholic acid; TCA, taurine-conjugated cholic acid; TCDCA, taurochenoxycholic acid; TDCA, taurodeoxycholic acid; TLCA, taurolithocholic acid; TUDCA, tauroursodeoxycholic acid; UDCA, ursodeoxycholic acid. **(b)** Representative bile acid tandem MS mass spectra used in quantification in the established assay. Glycine-conjugated cholic acid (GCA) and taurine-conjugated cholic acid (TCA) were shown as examples.

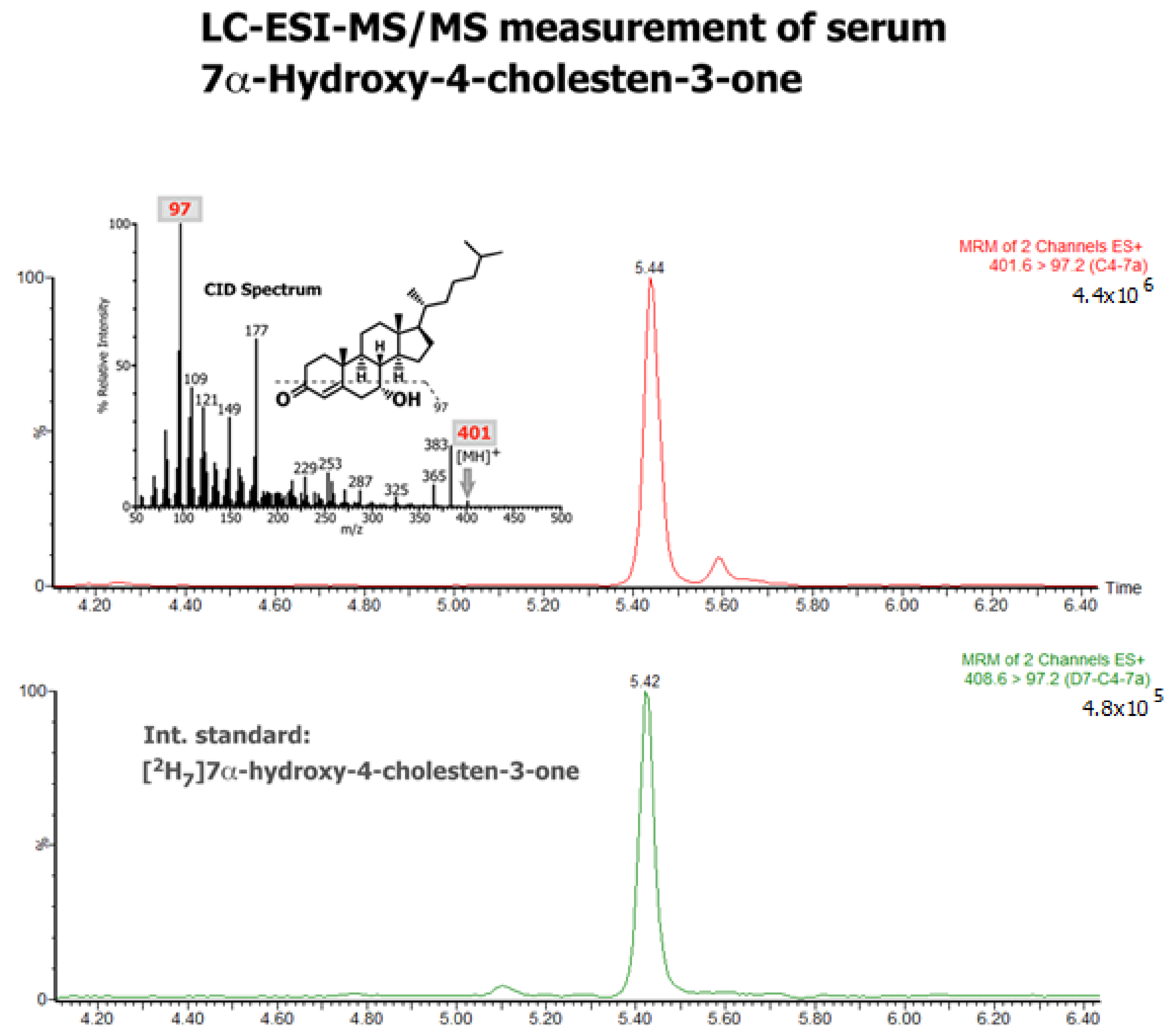

Supplemental Figure S2. Representative chromatogram of serum C4 profiling by UHPLC-MS/MS

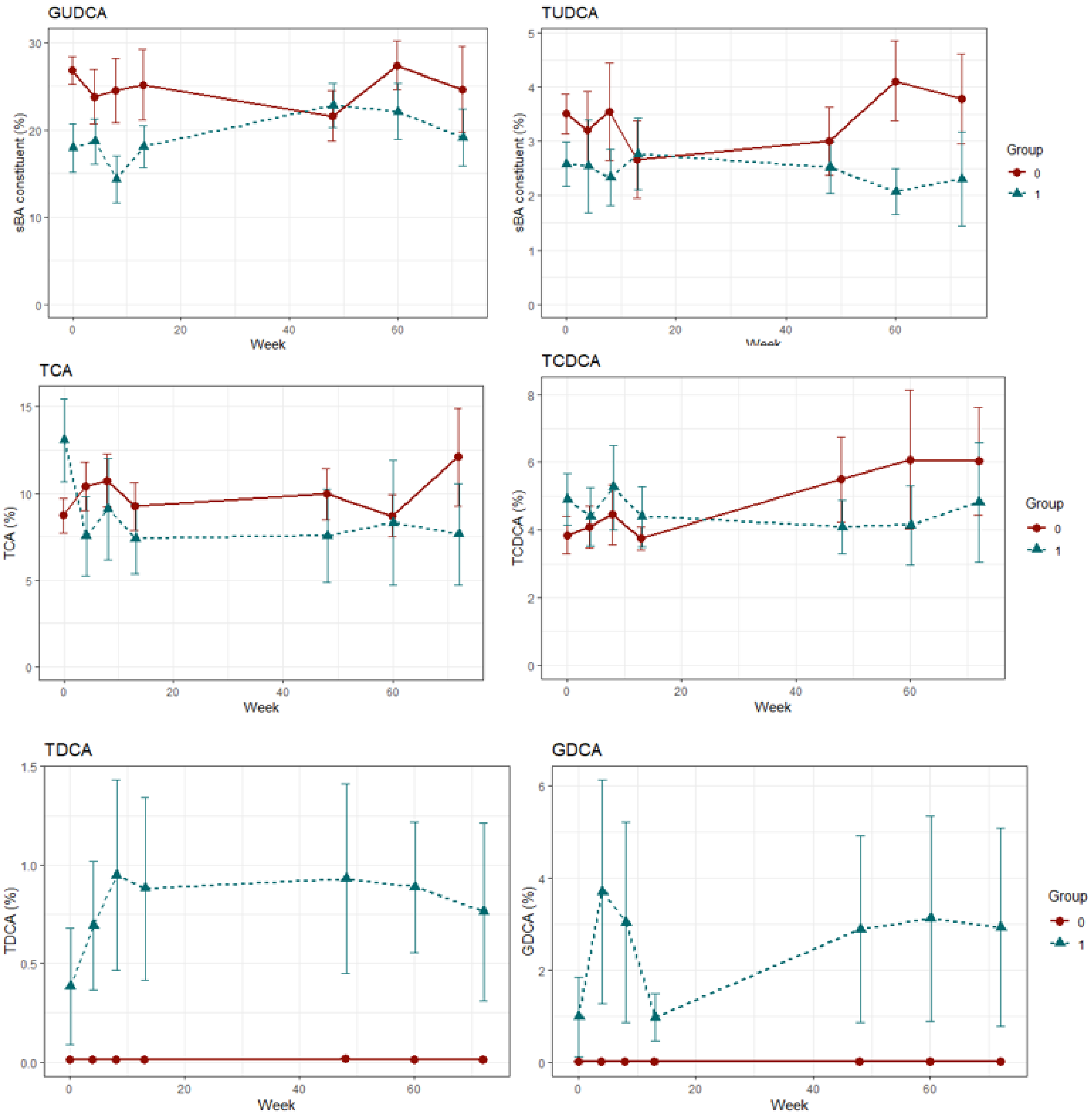

Supplemental Figure S3. Change in conjugated serum bile acid composition (%) over 72 weeks of maralixibat treatment in pruritus responders vs non-responders. Group 0-pruritus nonresponder, 1-pruritus responder

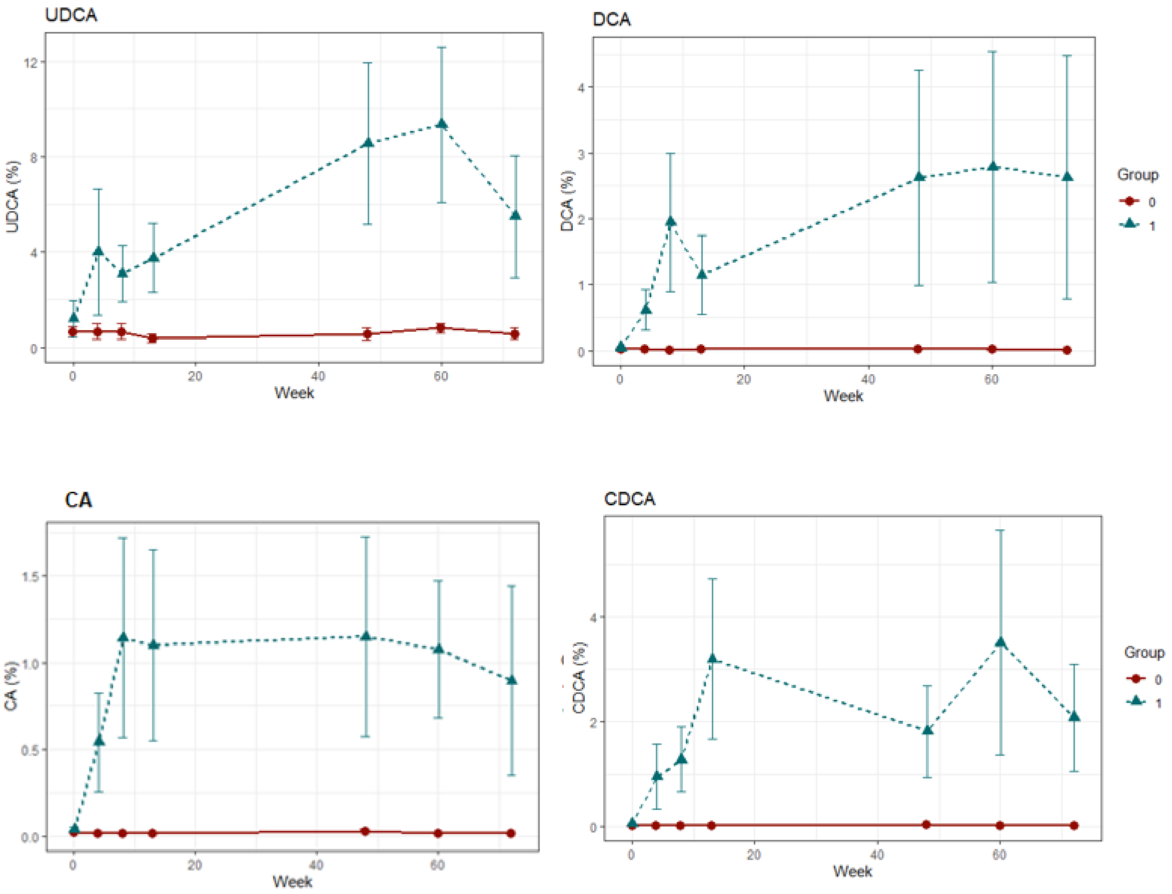

Supplemental Figure S4. Change in unconjugated serum bile acid composition (%) over 72 weeks of maralixibat treatment in pruritus responders vs non-responders. Group 0-pruritus nonresponder, 1-pruritus responder

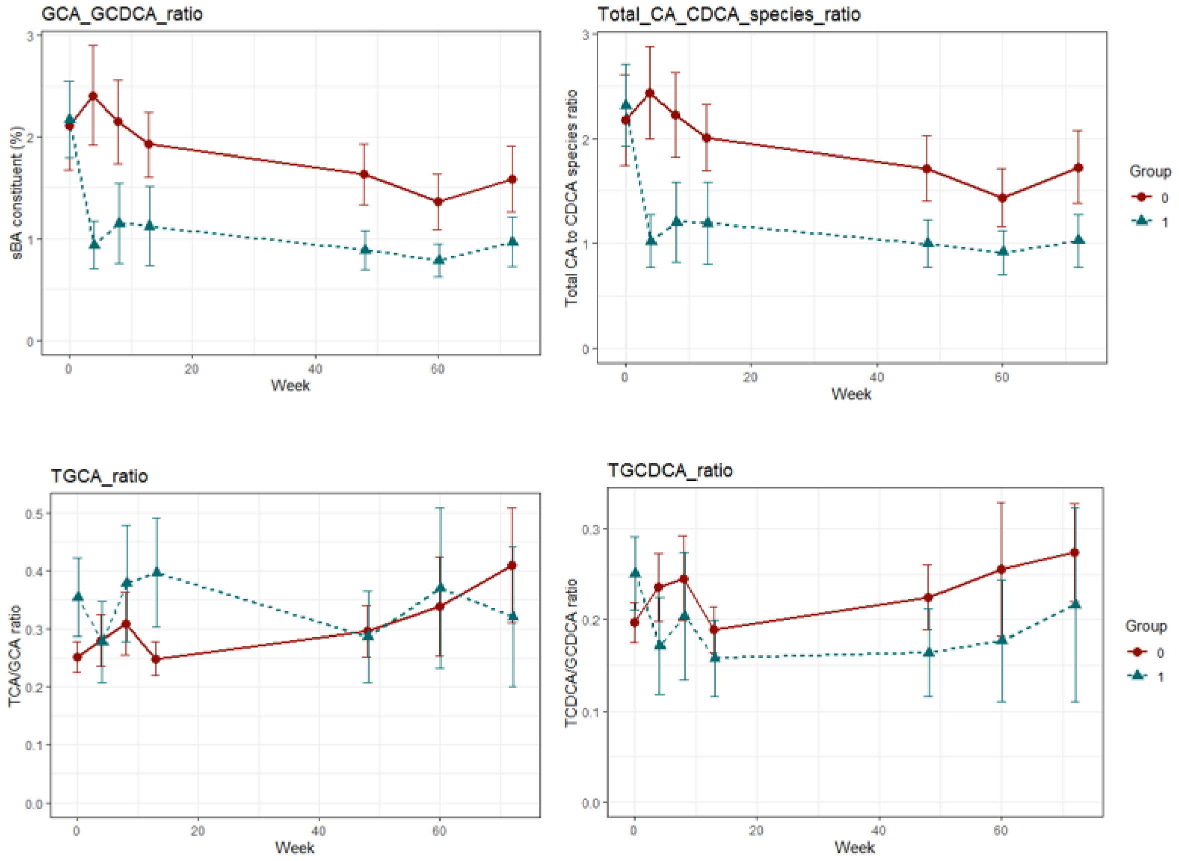

Supplemental Figure S5. Change in serum bile acid composition (%) relationship (ratio) over 72 weeks of maralixibat treatment in pruritus responders vs non-responders. Group 0-pruritus nonresponder, 1-pruritus responder

A

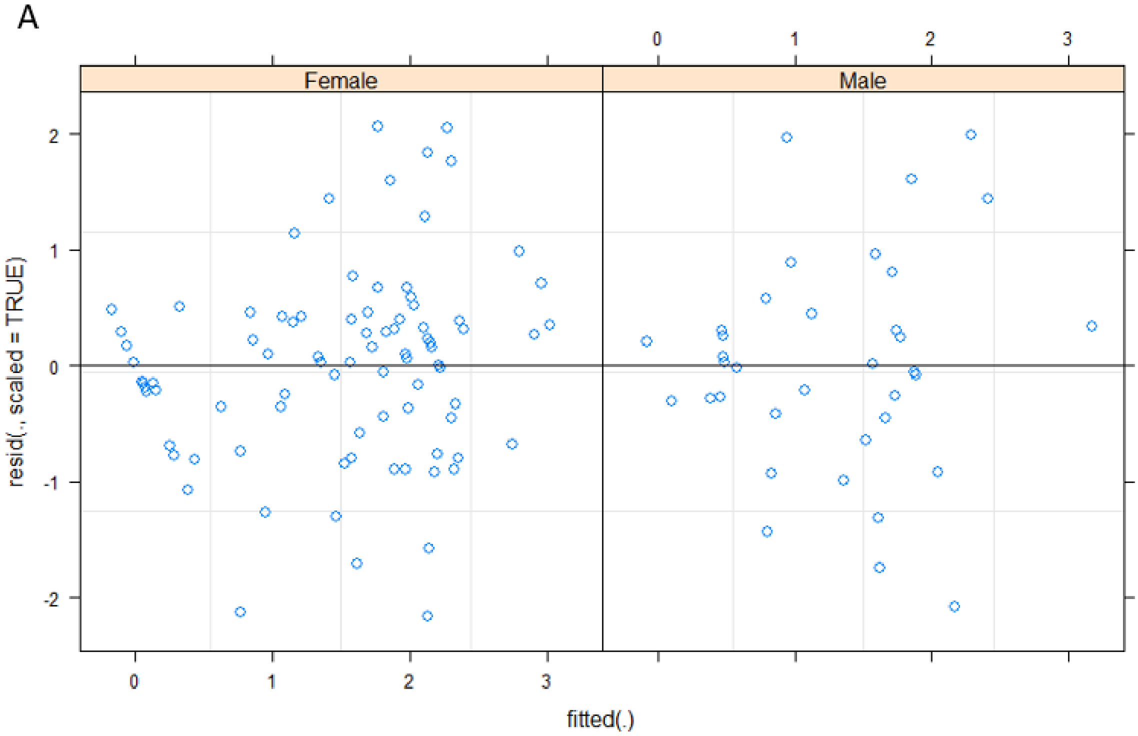
Female
Male
resid(., scaled = TRUE)
fitted(.)
2
1
0
-1
-2
0
1
2
3

B

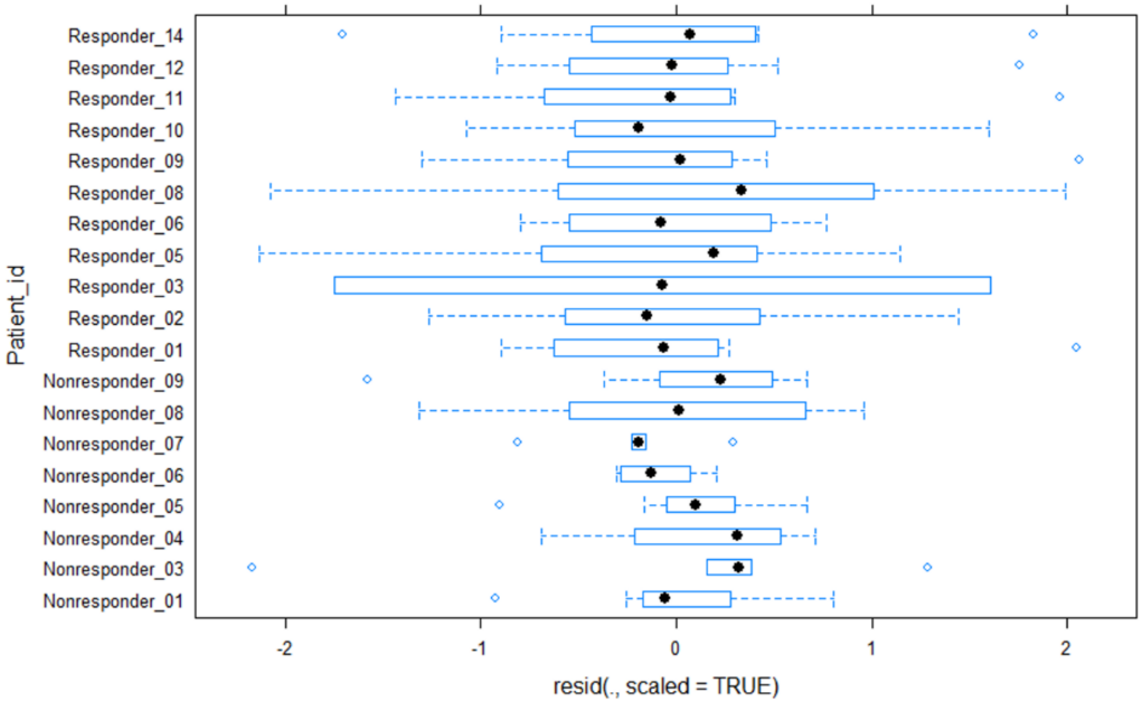
Patient_id
Responder_14
Responder_12
Responder_11
Responder_10
Responder_09
Responder_08
Responder_06
Responder_05
Responder_03
Responder_02
Responder_01
Nonresponder_09
Nonresponder_08
Nonresponder_07
Nonresponder_06
Nonresponder_05
Nonresponder_04
Nonresponder_03
Nonresponder_01
-2
-1
0
1
2
resid(., scaled = TRUE)

C

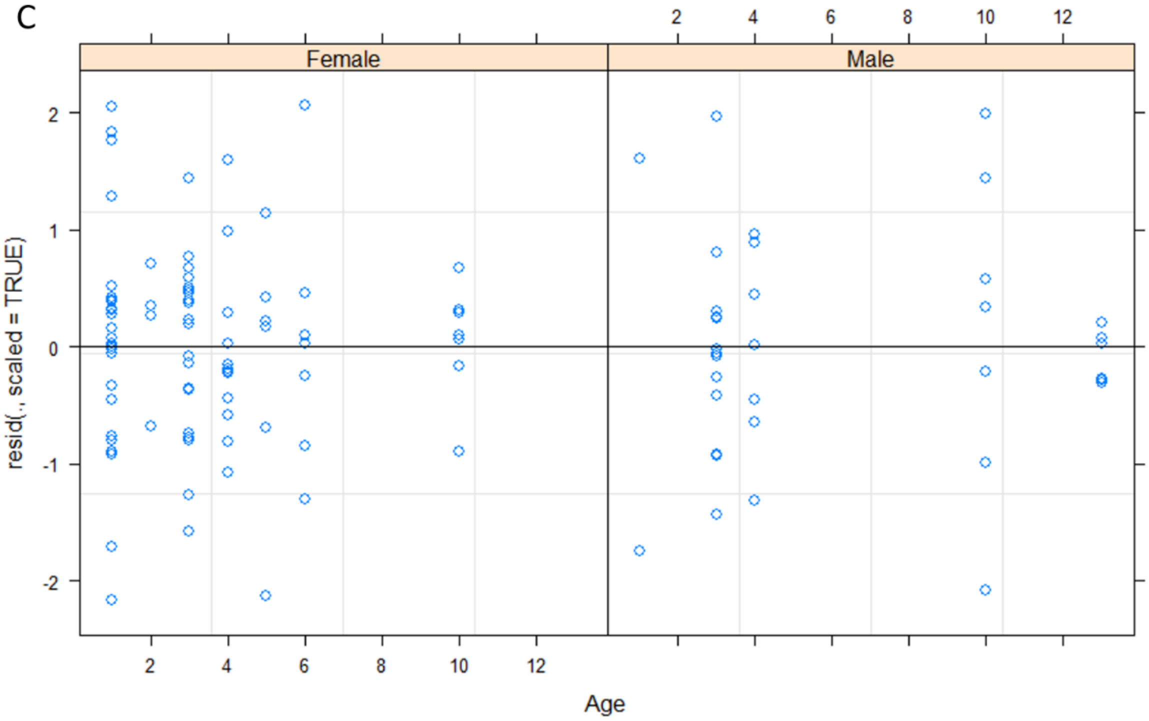

D

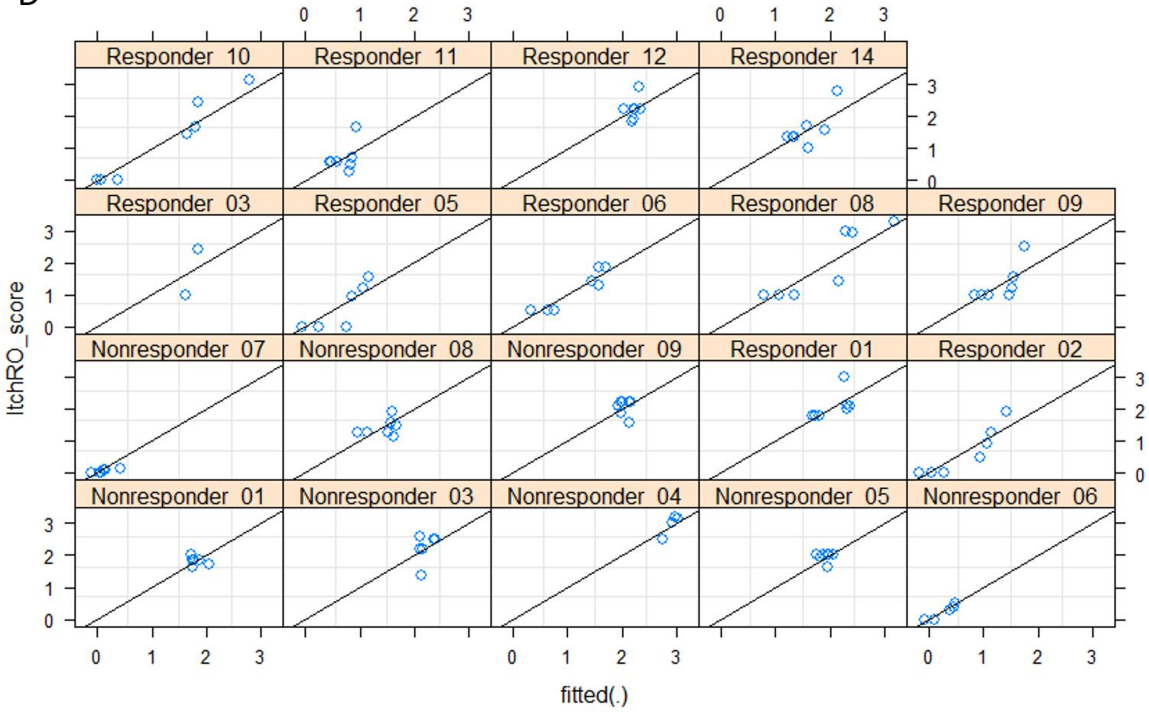

Supplemental Figure S6. (**A**) Residuals from LMM model plotted by gender; (**B**) LMM model residuals from each patient plotted based on pruritus responders vs non-responders; (**C**) Residuals from LMM model plotted by gender and age; (**D**) LMM model fitted outcome variable from each patient plotted comparatively to original data

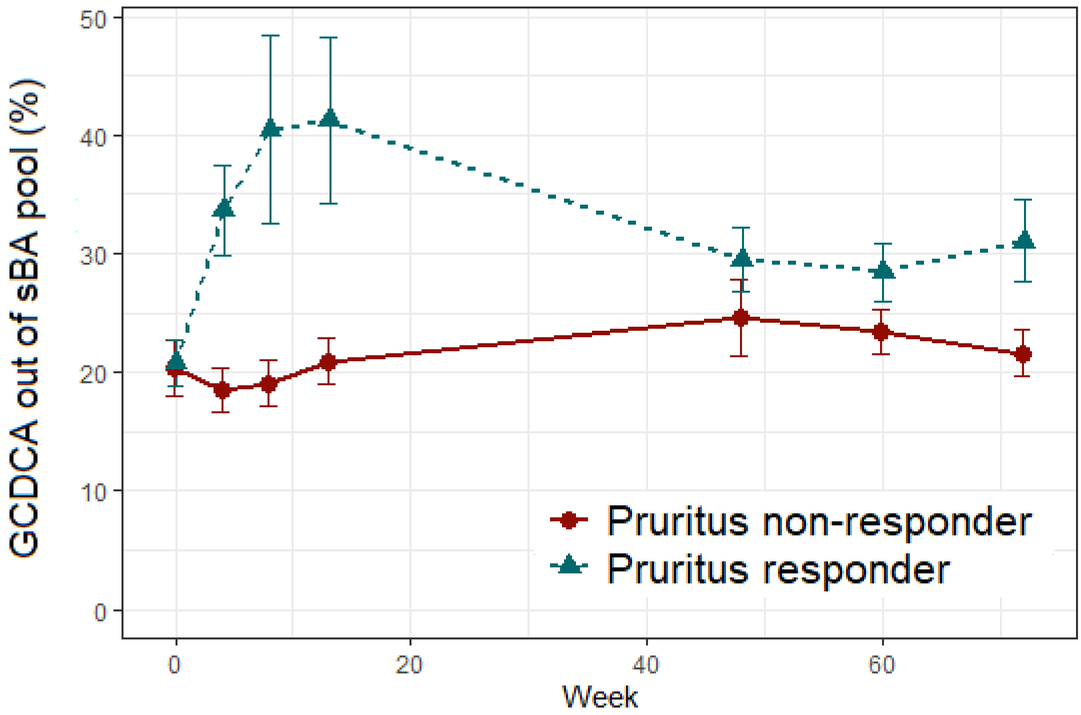

Supplemental Figure S7. Profile of GCDCA composition (%) over 72 weeks in PFIC2 patients under MRX treatment
