# Supplementary material for: Serum Bile Acid Profiling and Mixed Model Analysis Reveal Biomarkers Associated with Pruritus Reduction in Maralixibat-Treated Patients with BSEP Deficiency
Source: Metabolites. 2022 Oct 6;12(10):952. doi: 10.3390/metabo12100952 (PMC9609791; doi:10.3390/metabo12100952)
Supplement: Supplementary file 1 [file metabolites-12-00952-s001.zip › Supplemental_tables.pdf]

Supplemental Table S1. Levels of serum bile acid in patients with non-truncating BSEP following 72 weeks of maralixbat treatment compared with baseline

BA Hydrophobicity	BA species	Nonresponders			Responders		
		Baseline conc. (μM; n=8)	Week 72 conc. (μM; n=6)	p-value[a]	Baseline conc. (μM; n=11)	Week 72 conc. (μM; n=9)	p-value[a]
Hydrophilic	TCA	36.80 ± 19.87	60.48 ± 29.46	NS	48.38 ± 35.60	29.07 ± 49.14	NS
	TUDCA	14.63 ± 6.88	22.26 ± 15.07	NS	10.16 ± 8.25	6.70 ± 15.32	NS
	TCDCA	16.15 ± 10.30	34.62 ± 32.67	NS	18.14 ± 14.03	17.27 ± 32.10	NS
	GCA	144.64 ± 59.79	154.33 ± 41.41	NS	134.47 ± 82.70	64.06 ± 71.16	0.06
	GUDCA	108.72 ± 38.62	140.63 ± 83.33	NS	63.80 ± 57.55	28.19 ± 27.91	0.09
	GCDCA	81.87 ± 42.15	119.62 ± 57.63	NS	71.29 ± 47.69	49.15 ± 45.59	NS
	CA	0.08 ± 0.05	0.08 ± 0.04	NS	0.08 ± 0.04	0.06 ± 0.00	NS
	UDCA	2.13 ± 2.18	2.74 ± 2.46	NS	2.20 ± 3.18	1.17 ± 2.11	NS
	CDCA	0.06 ± 0.00	0.07 ± 0.02	NS	0.06 ± 0.00	0.13 ± 0.09	0.06
	TOTAL	405.09 ± 134.96	534.83 ± 155.37	NS	348.58 ± 183.29	195.79 ± 202.11	0.098
Hydrophobic	TDCA	0.05 ± 0.00	0.06 ± 0.02	NS	0.62 ± 1.21	0.12 ± 0.17	NS
	TLCA	0.05 ± 0.00	0.06 ± 0.01	NS	0.05 ± 0.00	0.05 ± 0.00	NS
	GDCA	0.06 ± 0.00	0.07 ± 0.03	NS	1.34 ± 3.02	0.23 ± 0.36	NS
	GLCA	0.06 ± 0.00	0.07 ± 0.02	NS	0.06 ± 0.00	0.06 ± 0.00	NS
	DCA	0.06 ± 0.00	0.07 ± 0.02	NS	0.07 ± 0.02	0.13 ± 0.18	NS
	LCA	0.06 ± 0.00	0.07 ± 0.02	NS	0.06 ± 0.00	0.06 ± 0.00	NS
	TOTAL	0.35 ± 0.00	0.41 ± 0.10	NS	2.20 ± 4.19	0.65 ± 0.49	NS

[a]$p < 0.10$ was considered to be statistically significant given dataset limitations

BA, bile acid; BSEP, bile salt export pump; CA, cholic acid; CDCA, chenodeoxycholic acid; DCA, deoxycholic acid; LCA, lithocholic acid; GCA, glycine-conjugated cholic acid; GCDCA, glycochenodeoxycholic acid; GDCA, glycodeoxycholic acid; GLCA, glycolithocholic acid; GUDCA, glycoursodeoxycholic acid; NS, non-significant; TCA, taurine-conjugated cholic acid; TCDCA, taurochenoxycholic acid; TDCA, taurodeoxycholic acid; TLCA, taurolithocholic acid; TUDCA, tauroursodeoxycholic acid; UDCA, ursodeoxycholic acid

Data are mean ± standard deviation. The two-tailed Student's t-test was used to compare groups.

Supplemental Table S2. Total BA as biomarker – model optimization

Fix effect	Random effect	AIC	BIC	Random effect residual variance
1 + Week + Age + Gender + Predictor_baseline +Total_BA	1 + Week ǀ Subject_ID	203.1	231.0	0.1279
1 + Week + Gender + Predictor_baseline +Total_BA	1 + Week ǀ Subject_ID	200.3	225.4	0.1278
1 + Week + Age + Predictor_baseline +Total_BA	1 + Week ǀ Subject_ID	200.1	225.3	0.1279
1 + Week + Age + Gender + Total_BA	1 + Week ǀ Subject_ID	201.1	226.3	0.1278

Supplemental Table S3. Likelihood-ratio test for reduced model excluding age, gender, and pre-dose baseline vs. full model

LMM model	npar	AIC	BIC	logLik	deviance	Chisq	Df	Pr(>Chisq)
`ItchRO Weekly Average Score` ~ 1 + Week + Total_BA + (1 + Week ∣ Subject_ID)	7	197.81	217.38	−91.905	183.81			
`ItchRO Weekly Average Score` ~ 1 + Week + Age + Predictor_baseline + Gender + Total_BA + (1 + Week ∣ Subject_ID)	10	202.06	230.02	−91.030	182.06	1.751	3	0.6257

Supplemental Table S4. Summary of linear mixed model (LMM) of pruritus ItchRO score ~ UDCA (%)

		**ItchRO_score**	
**Predictors**	**Estimates**	CI	p-value
(Intercept)	1.79	1.46 – 2.12	**< 0.001**
Week	-0.01	−0.01 – −0.00	**0.001**
UDCA	-0.03	−0.05 – −0.01	**0.002**
**Random Effects**			
σ^2		0.14	
τ_{00} Patient_id		0.47	
τ_{11} Patient_id.Week		0.00	
ρ_{01} Patient_id		−0.13	
ICC		0.80	
N Patient_id		19	
Observations		121	
Marginal R^2 / Conditional R^2		0.139 / 0.828	

*Significant p-values, i.e. at 0.05 level, are highlighted in bold.

Supplemental Table S5. Summary of linear mixed model (LMM) of pruritus ItchRO score ~ GCA (%)

		ItchRO_score	
**Predictors**	**Estimates**	**CI**	**p-value**
(Intercept)	0.97	0.54 – 1.40	**< 0.001**
Week	-0.01	−0.01 – −0.00	**0.006**
GCA	0.02	0.01 – 0.03	**< 0.001**
**Random Effects**			
σ^2		0.12	
τ_{00} Patient_id		0.45	
τ_{11} Patient_id.Week		0.00	
ρ_{01} Patient_id		−0.33	
ICC		0.81	
N Patient_id		19	
Observations		121	
Marginal R^2 / Conditional R^2		0.229 / 0.853	

*Significant p-values, i.e. at 0.05 level, are highlighted in bold.

Supplemental Table S6. Summary of linear mixed model (LMM) of pruritus ItchRO score ~ TCA (%)

	ItchRO_score		
**Predictors**	**Estimates**	**CI**	**p-value**
(Intercept)	1.32	0.93 – 1.71	**<0.001**
Week	-0.01	−0.02 – −0.00	**<0.001**
TCA	0.04	0.02 – 0.06	**<0.001**
**Random Effects**			
σ^2	0.14		
τ_{00} Patient_id	0.44		
τ_{11} Patient_id.Week	0.00		
ρ_{01} Patient_id	−0.37		
ICC	0.76		
N Patient_id	19		
Observations	121		
Marginal R^2 / Conditional R^2	0.226 / 0.815		

*Significant p-values, i.e. at 0.05 level, are highlighted in bold.

Supplemental Table S7. Summary of general linear mixed model (GLMM) of pruritus responder class ~ GCA (%)

	Pruritus responder		
**Predictors**	**Odds Ratios**	**CI**	**p-value**
(Intercept)	0.00	0.00 – 973.31	0.274
Week	0.93	0.74 – 1.16	0.527
GCA	0.88	0.58 – 1.33	0.548
**Random Effects**			
σ^2	3.29		
$\tau_{00\ Patient\_id}$	375.26		
$\tau_{11\ Patient\_id.Week}$	24.83		
$\rho_{01\ Patient\_id}$	−0.84		
ICC	1.00		
$N_{Patient\_id}$	19		
Observations	121		
Marginal R^2 / Conditional R^2	0.000 / 1.000		

Supplemental Table S8. Summary of general linear mixed model (GLMM) of pruritus responder class ~ ratio of total cholic acids/total chenodeoxycholic acids

	Pruritus_responding		
**Predictors**	**Odds Ratios**	**CI**	**p-value**
(Intercept)	0.00	0.00 – 0.75	**0.044**
Week	0.94	0.75 – 1.17	0.564
Total CA/CDCA ratio	0.22	0.01 – 8.12	0.411
**Random Effects**			
σ^2	3.29		
τ_{00} Patient_id	429.46		
τ_{11} Patient_id.Week	27.69		
ρ_{01} Patient_id	−0.85		
ICC	1.00		
N Patient_id	19		
Observations	121		
Marginal R^2 / Conditional R^2	0.000 / 1.000		

*Significant p-values, i.e. at 0.05 level, are highlighted in bold.
